# YAP Promotes Ovarian Cancer Cell Tumorigenesis and Is Indicative of a Poor Prognosis for Ovarian Cancer Patients

**DOI:** 10.1371/journal.pone.0091770

**Published:** 2014-03-12

**Authors:** Yan Xia, Ting Chang, Yingmei Wang, Yixiong Liu, Wenhui Li, Ming Li, Heng-Yu Fan

**Affiliations:** 1 Life Sciences Institute and Innovation Center for Cell Biology, Zhejiang University, Hangzhou, China; 2 Department of Neurology, Tangdu Hospital, the Fourth Military Medical University, Xi'an, China; 3 Department of Pathology, Xijing Hospital, the Fourth Military Medical University, Xi'an, China; 4 Department of Obstetrics and Gynecology, Xijing Hospital, the Fourth Military Medical University, Xi'an, China; 5 The Affiliated Guangren Hospital, Xi'an Jiaotong University, Xi'an, China; Institute of Zoology, Chinese Academy of Sciences, China

## Abstract

YAP is a key component of the Hippo signaling pathway and plays a critical role in the development and progression of multiple cancer types, including ovarian cancer. However, the effects of YAP on ovarian cancer development *in vivo* and its downstream effectors remain uncertain. In this study we found that strong YAP expression was associated with poor ovarian cancer patient survival. Specifically, we showed for the first time that high YAP expression levels were positively correlated with TEAD4 gene expression, and their co-expression was a prognostic marker for poor ovarian cancer survival. Hyperactivation of YAP by mutating its five inhibitory phosphorylation sites (YAP-5SA) increased ovarian cancer cell proliferation, resistance to chemotherapeutic drugs, cell migration, and anchorage-independent growth. In contrast, expression of a dominant negative YAP mutant reversed these phenotypes in ovarian cancer cells both *in vitro* and *in vivo*. Our results suggested that YAP caused these effects by promoting an epithelial-to-mesenchymal transition. Thus, YAP promotes ovarian cancer cell growth and tumorigenesis both *in vitro* and *in vivo*. Further, high YAP and TEAD4 expression is a prognostic marker for ovarian cancer progression and a potential target for ovarian cancer treatment.

## Introduction

Epithelial ovarian cancers (EOCs) comprise the majority of malignant ovarian tumors in adult women and have the worst prognosis among female cancers based on their 5-year survival rates. These patients often present with non-specific pelvic or abdominal symptoms. Conventional treatments usually include surgery, platinum and Taxol-based chemotherapies, and radiation therapy. Due to the lack of specific symptoms and effective screening methods, patients are often diagnosed when at an advanced stage and their prognosis is even worse than with other cancers.

The Hippo pathway is a recently discovered signal transduction pathway. The core components of the Hippo pathway, including MST1/2, LATS, Sav1, and YAP, are highly conserved from the fruit fly (*Drosophila*) to mammals [1]–[6]. MST1/2 activation results in the phosphorylation and activation of their direct substrates LATS1/2. Activated LATS1/2, in turn, phosphorylate and inhibit YAP and TAZ transcription co-activator [7], [8]. YAP promotes cell proliferation, inhibits cellular apoptosis, and also promotes an epidermal-mesenchymal transition (EMT) [9]–[12]. YAP is closely associated with tumorigenesis as a critical transcription co-activator in the Hippo pathway.

Several transcription factors, including ErbB4, RUNX2, TEAD family members, and p73, have been found to be YAP downstream target genes [13]–[16]. Because it lacks a DNA binding domain, YAP binds to the C-terminal region of TEAD in order to regulate downstream genes' transcription and translation. Zhao *et al* identified TEAD family transcription factors as the most potent YAP targets and TEAD family members were important for the growth-promoting function of YAP [14], [17]–[21].

Previous studies showed that YAP was highly expressed in human ovarian cancer tissues and that high YAP activity promoted the proliferation and survival of cultured ovarian cancer cells[9], [22]. However, the role of YAP in ovarian cancer development *in vivo* and the association of YAP/TEAD with ovarian cancer patient survival have not been thoroughly investigated.

In this study we show that continuous YAP activation induces increased ovarian cancer cell proliferation, resistance to cisplatin-induced cellular apoptosis, loss of contact inhibition, increased cell migration, and anchorage-independent growth both *in vitro* and *in vivo*. Consistent with these results, we also found that high YAP expression was associated with poor ovarian cancer patient survival. Further, TEAD family members were also expressed in ovarian cancer tissues. Interestingly, YAP expression was positively correlated with TEAD4 expression and their co-expression was closely associated with poor ovarian cancer patient survival. Taken together, these findings indicate that YAP is a key ovarian cancer oncogene and that YAP/TEAD4 co-expression may be a predictor of a poor prognosis for human ovarian cancer.

## Materials and Methods

### Nude mice xenografts and *In Vivo* treatments

Nude mice were obtained from the Center of Experimental Animals, Zhejiang University.

Mice were treated in accordance with the NIH Guide for the Care and Use of Laboratory Animals, approved by ethics committee of Zhejiang University. Mice were housed in a temperature-controlled room with proper darkness-light cycles, fed with a regular diet, and maintained under the care of the Laboratory Animal Unit, Zhejiang University, China. The ovarian cancer cell-transplanted mice were examinined daily, and were euthanized using CO2 inhalation method before being sacrificed. To examine the effects of YAP activity on tumors *in vivo*, stable cell lines that expressed YAP-5SA or dominant negative YAP were cultured and used to induce tumors by implanting 2×10^6^ cells in 100 μl of PBS subcutaneously into the right abdominal flanks of 4–6 week old female nude mice. To determine if YAP has effect on the chemo-resistance, A2780CP YAP-5SA-ΔC cells and control cells (2×10^6^ cells per mouse in each group) were separately inoculated (s.c.) into nude mice. After tumors grew to about 5 mm in diameter, 3.5 mg/body weight (kg)/day of CDDP was administered (i.p.) to the nude mice once a week for 4 weeks. Tumor sizes (both longitudinal and transverse widths) were measured with a caliper.

### Tissue array and IHC Analysis

Formalin-fixed and paraffin-embedded ovarian cancer tissue array was obtained from US Biomax, Inc. (Rockville, MD). The tissue microarray was immunohistochemically stained for YAP (#4912, Cell Signaling), S127 phosphorylated-YAP (#4911S, Cell Signaling), TEAD1 (5178-1, Epitomics), TEAD2 (LS-C119063, Lifespan Biosciences),TEAD3 (LS-C30406, Lifespan Biosciences),and TEAD4 (ab97460, Abcam). In addition, 45 human ovarian cancer specimens for patient follow-up are from the Department of Pathology, Xijing Hospital of Fourth Military Medical University. The ovarian cancer specimen use and the study protocol were approved by the Ethics Committee of Xijing Hospital, and written informed consent was obtained from each patient.

All the final score of each sample (negative or positive) was scored independently by two pathologists and assessed by adding the scores for the intensity and extent of staining. The intensity of staining was scored as 0 (negative), 1 (weak), or 2 (strong). The extent of staining was scored based on the percentage of positive tumor cells: 0 (negative), 1 (1%–30%), 2 (30%–60%), and 3 (61%–100%). Each case was finally considered negative if the final score was 0 (negative) or 1 (weak positive) and positive if the final score was 2 to 3 (+) or 4 to 5 (strong positive).

### Cell lines

All the cell lines used in this study were originally purchased from ATCC. All the cell culture media were from Invitrogen. SKOV-3 cells were cultured in McCoy's 5A medium with 10% FBS. A2780 cells were grown in DMEM High Glucose medium supplemented with 10% FBS and a 1% penicillin-streptomycin solution. OVCAR3 cells were cultured in DMEM medium with 0.01 mg/ml of insulin and 15% FBS. OVCAR8 cells were grown in RPMI 1640 medium with 10% FBS. All cell lines were cultured with 10 units/ml of penicillin and 10 ng/ml of streptomycin. Stable cell lines were grown in culture medium and selected using different concentrations of puromycin.

### Cell proliferation assay

For each condition described above, cells were cultured in triplicate wells in 96 well plates at 2000 cells/well. Cell growth was determined by measuring the absorbance with a plate reader (Bio-Rad) after culture for 24 hours. Three independent experiments were done for each treatment.

### Western Blot analysis

Cell extracts containing 30 μg of protein were resolved by SDS-PAGE and transferred to PVDF membranes (Millipore Corp., Bedford, MA). After probing with primary antibodies, the membranes were incubated with horseradish peroxidase-linked anti-rabbit antibodies (Cell Signaling Technologies, Danvers, MA) and then washed. Bound antibodies were visualized using an Enhanced Chemiluminescence Detection Kit (Amersham). The primary antibodies were: YAP (#4912), S127 phosphorylated-YAP (#4911S), PARP (#9542), cleaved caspase-3 (#9664), β-actin (#AC40), E-cadherin (#3195), Slug (#3195), and Snail (#3895) from Cell Signaling. A TEAD1 (5178-1) antibody was from Epitomics. TEAD2 (LS-C119063) and TEAD3 (LS-C30406) antibodies were from Lifespan Biosciences. A TEAD4 (ab97460) antibody was from Abcam.

### Flat colony and soft agar colony forming assays

Cells were plated in triplicate wells in 6-well plates for 14 days for a flat colony formation assay. For a soft agar assay, 2,000 cells were plated in complete medium plus 0.6% agar in triplicate wells in 6-well plates. Medium was replaced every 48 hours and visible colonies were counted by light microscopy after 21 days.

### Scratch assay

Cells were plated in 6-well plates and cultured in serum-free medium to block proliferation. After 12 hours, cells were scratched using a 200 μl pipette tip. Then, images of 10 scratches per cell line were acquired at 0 h, 12 h, and 24 h.

### Apoptosis assay

Cells were cultured in medium with different concentrations of cisplatin for 48 hours. Then, cells were harvested with an apoptosis detection kit (BD Biosciences, 559763) by flow cytometry and for Western blot analysis.

### *In vitro* and *in vivo* metastatic model and bioluminescent imaging

A 24-well transwell plate (8-μm pore size, Corning, USA) was used to determine the migration and invasive capability of each cell line. For transwell migration assays, 5×10^4^ cells were seeded in the top chamber that was lined with a non-coated membrane. The lower chambers were all added into 500 ul culture medium with 20% FBS. The mean values of triplicate assays for each experimental condition were determined.

For *in vivo* metastasis assays, HO8910 PM-YAP △C stable cells was infected with a lusiferase reporter plasmid and injected into the caudal veins of 4 week female null mice. After two weeks, the animals were imaged weekly by the animal imaging machine using an intensified charge coupled device (CCD) camera system. For *in vivo* bioluminescence imaging, mice were anesthetized using a 1–2% isofluorane/air mixture and injected with a single (i.p.) dose of 100 mg/kg of Luciferin (Promega, WI) and bioluminescence was detected with an IVIS 100 Imaging System (Xenogen).

Besides the bioluminescence imaging, mice were executed at 60 days after ovarian cancer cell-injection for investigating the post-inoculation organ metastases.

### Statistical analysis

For the 45 subjects with complete immunohistochemical and outcome data, disease-specific 5-year survival was estimated using the Kaplan-Meier method and compared between groups using log-rank tests. Correlation analysis was completed by Chi-square test. Student's t test was used to compare the results of each measured variable with control results. P-values of <0.05 were considered significant. All analyses were done using SPSS software (version 11.0).

## Results

### YAP is significantly upregulated in human ovarian cancer tissues and high YAP expression predicts poor patient prognosis

**Figure 1:**
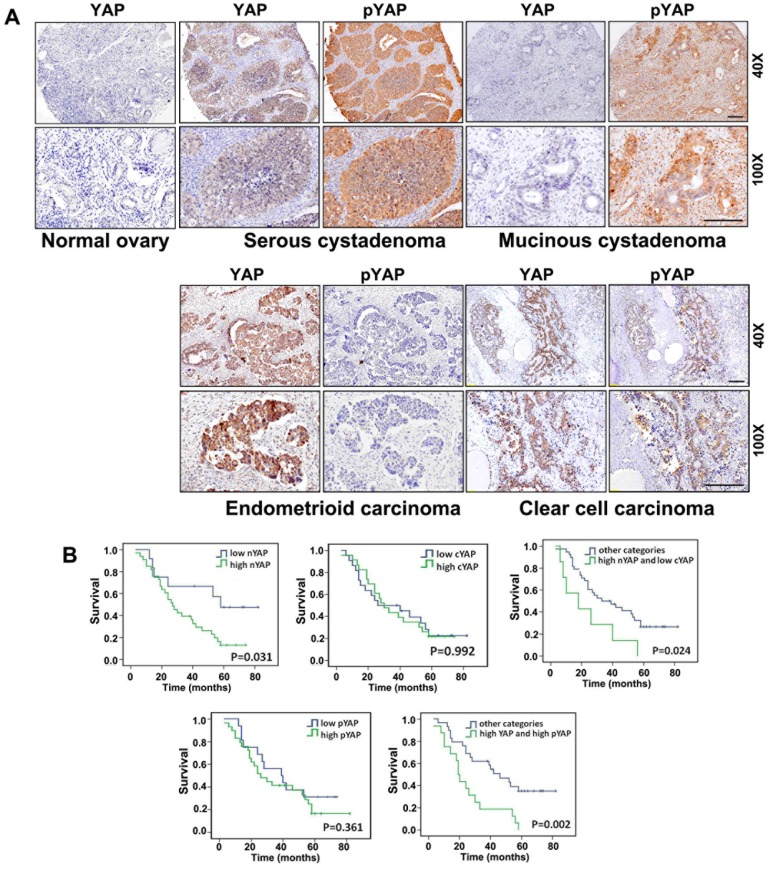
YAP is significantly upregulated in human ovarian cancer tissues and is associated with patient prognosis. **A**: Immunohistochemistry results for YAP and pYAP expression in normal human ovary tissues and four subtypes of ovarian cancer tissue. Sections were counterstained with hematoxylin. Magnifications at 40× and 100× are shown. Scale bar  = 100 μM for all panels. **B**: Kaplan-Meier analysis for associations between YAP expression, localization, and phosphorylation and ovarian cancer patient survival. P-values are from log-rank tests.

To investigate if YAP was upregulated in human ovarian cancer, total and phosphorylated YAP (pYAP) were detected by immunohistochemistry (IHC) and slides were evaluated based on cytoplasmic and nuclear YAP staining levels. These IHC sections were categorized as having either negative, low, or high staining. As a control, 10 normal ovarian tissue sections were immunohistochemically stained with YAP. However, YAP expression was not detected in these sections (Figure 1A).

YAP expression and subcellular localization varied for different tumor types (Figure 1A). High nuclear YAP levels were observed for 22.64% of the 106 ovarian tumor samples analyzed, which included 9 metastatic ovarian cancer samples, whereas pYAP was observed in 11.88% of tumor samples. Of 24 samples with strong YAP IHC signals, 13 were also strongly positive for pYAP. Among the four major types of ovarian cancers, YAP was strongly expressed in serous and endometrioid cancinoma samples (86.67%; N = 15).

To determine if YAP and pYAP distributions were associated with ovarian cancer patient survival, YAP and pYAP localization and expression levels were evaluated in 45 ovarian cancer pathological sections. These results were used for Kaplan-Meier analysis to estimate disease-specific survival. Both cytoplasmic YAP (cYAP) and pYAP alone were not associated with a poor prognosis for human ovarian cancer. However, when total YAP expression levels were taken into account, these were significantly associated with a poor prognosis (Figure 1B). These results suggested that high YAP expression levels rather than its subcellular distributions were associated with ovarian cancer patient survival.

### YAP promotes ovarian cancer cell proliferation and tumorigenesis

**Figure 2:**
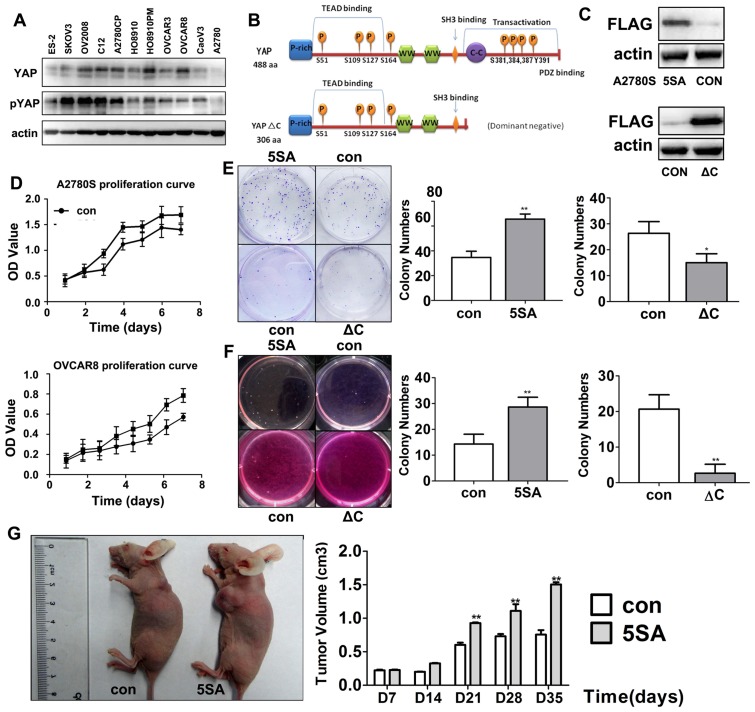
YAP promotes human ovarian cancer cell proliferation and tumorigenesis. **A**: Western blotting results for endogenous YAP expression in 11 ovarian cancer cell lines. **B**: Diagram of the main functional structural domains and phosphorylation sites in human YAP (full length and C-terminal deleted, YAP-ΔC, forms). **C**: Western blotting results for the expression of FLAG-tagged YAP-5SA and YAP-5SA-ΔC in established stable cell lines. **D**: Growth curves for cells that stably expressed YAP-5SA (upper panel) and YAP-5SA-ΔC (lower panel) and their control cells during a 7-day culture period. **E**: Images and quantitative results for flat plate colony formation assays. Cells that expressed YAP-5SA or YAP-5SA-ΔC and their control cells were cultured in 6-well plates for 2 weeks. **F**: Images and quantitative results for colonies grown in soft agar. Cells that expressed YAP-5SA or YAP-5SA-ΔC and their control cells were seeded in soft agar for 3 weeks. **G**: Images and quantitative results for xenografts grown in nude mice. Mice were subcutaneously injected with YAP-5SA expressing and control cells. Each point is the mean of 3 experiments. Error bars represent s.d. 's; n = 5. Statistically significant differences as compared with a control as determined by Student's t-test are denoted by *(P<0.05) or **(P<0.01).

In addition to human ovarian cancer samples, we also determined endogenous YAP expression in 11 cultured ovarian cancer cell lines. Western blotting results showed that YAP was highly expressed in most of these cell lines, with the weakest expression in A2780 cells and the highest expression in OVCAR8 cells (Figure 2A). Thus, these two cell lines were used for the following experiments.

To investigate a role for YAP in ovarian cancer cell proliferation and metastasis, we generated A2780 and OVCAR8 stable cell lines that overexpressed constitutively active YAP (YAP with five LATS1/2 phosphorylation site mutations; YAP-5SA) and dominant negative YAP (YAP-5SA with a C-terminal transactivation domain deletion; YAP-5SA-ΔC; Figure 2B-C). Cell growth curves showed that YAP-5SA expressing cells had greater proliferative activity than the controls (Figure 2D, upper panel). In contrast, YAP-5SA-ΔC expressing OVCAR8 cells had a decreased proliferation rate relative to control cells (Figure 2D, lower panel).

Next, we investigated if YAP activity affected cell transformation and migration in ovarian cancer cells using a plate colony formation assay. These results showed that constitutively active YAP-5SA promoted clone formation, but that dominant negative YAP-5SA-ΔC suppressed clone formation (Figure 2E). In addition, we used a soft agar assay to determine anchorage-independent growth, another indicator of tumourigenicity. As shown in Figure 2F, YAP activity was positively correlated with ovarian cancer cells' colony formation capability in soft agar.

### YAP promotes ovarian cancer cells proliferation and tumorigenesis *in vivo*

Previous studies did not investigate if YAP could enhance tumorigenesis *in vivo*. Thus, we subcutaneously injected YAP-5SA and YAP-5SA-ΔC expressing cells, as well as their respective control cells, into 4-week-old nude mice. After 3 weeks, the tumors derived from YAP-5SA stable cells were significantly larger than those of controls (Figure 2G). These results were consistent with our *in vitro* assays.

### YAP enhances ovarian cancer cell lines' resistance to Cisplatin and Taxol

**Figure 3:**
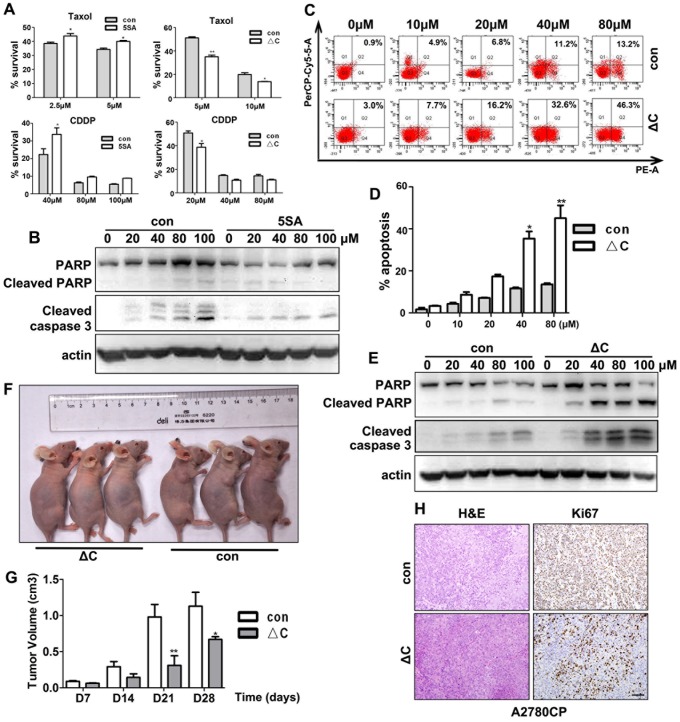
YAP enhances chemotherapeutic drug resistance by ovarian cancer cells. **A**: Viability of YAP-5SA and YAP-5SA-ΔC expressing cells after treatment with Taxol or CDDP, as assessed by MTT assay. **B**: Western blotting results for the apoptosis markers cleaved caspase 3 and PARP in YAP-5SA expressing and control cells after treatment with the indicated doses of CDDP for 48 h. **C**: Flow cytometry results for apoptosis of A2780CP cells with or without YAP-5SA-ΔC transfection and after treatment with different doses of CDDP for 48 h. **D**: Western blotting results for cleaved caspase 3 and PARP in YAP-5SA-ΔC expressing and control cells after treatment with the indicated doses of CDDP for 48 h. **F-G**: Images and quantitative results for *in vivo* tumorigenic capacity of A2780CP cells with or without YAP-5SA-ΔC expression. Nude mice were injected with CDDP through a caudal vein once each week for four weeks after tumor xenografts reached 5 mm in diameter. **H**: IHC results for the proliferation marker Ki67 on the indicated tumor tissue sections. Scale bar  = 100 μM.

Cisplatin and Taxol are the primary anti-cancer drugs used for ovarian cancer therapy. Thus, we sought to determine if YAP activity affected ovarian cancer cells' sensitivities to Cisplatin and Taxol. As shown in Figure 3A, YAP-5SA expressing cells were more resistant to both of these drugs than were control cells at different doses. In contrast, YAP-5SA-ΔC expressing cells were more susceptible to both drugs than were control cells. The apoptotic cell markers cleaved PARP and cleaved caspase 3 were detected in A2780 control cells after Cisplatin treatment in a dose-dependent manner (0–100 μM). However, the increased expressions of these apoptosis markers were less significant in similarly treated YAP-5SA expressing cells (Figure 3B).

Flow cytometry results showed that a previously reported cisplatin-resistant ovarian cancer cell line (A2780CP) was indeed resistant to cisplatin treatment at low doses (10–40 μM). However, YAP-5SA-ΔC expression in these cells caused an increase in cellular apoptosis after cisplatin treatment at all of the doses tested (Figure 3C–D). Moreover, YAP-5SA-ΔC expressing A2780CP cells showed remarkably increased expression of cleaved PARP and caspase 3 than did control cells after cisplatin treatment (Figure 3E).

To determine whether YAP conferred drug resistance to ovarian cancer cells *in vivo*, we subcutaneously injected nude mice with A2780CP cells that stably expressed YAP-5SA-ΔC and with control cells. At 1 week after cell injections, mice were treated with cisplatin by caudal vain injection every three days. Tumor sizes were measured at 5 weeks after ovarian cancer cell injections. Compared with controls, tumors that were derived from YAP-5SA-ΔC expressing cells were smaller in size compared to those derived from control cells (Figure 3F-G). Cell proliferation marker Ki67 expression was weaker in YAP-5SA-ΔC expressing cells than in control cells (Figure 3H).

### YAP enhances the migration and anchorage-independent growth of ovarian cancer cells

**Figure 4:**
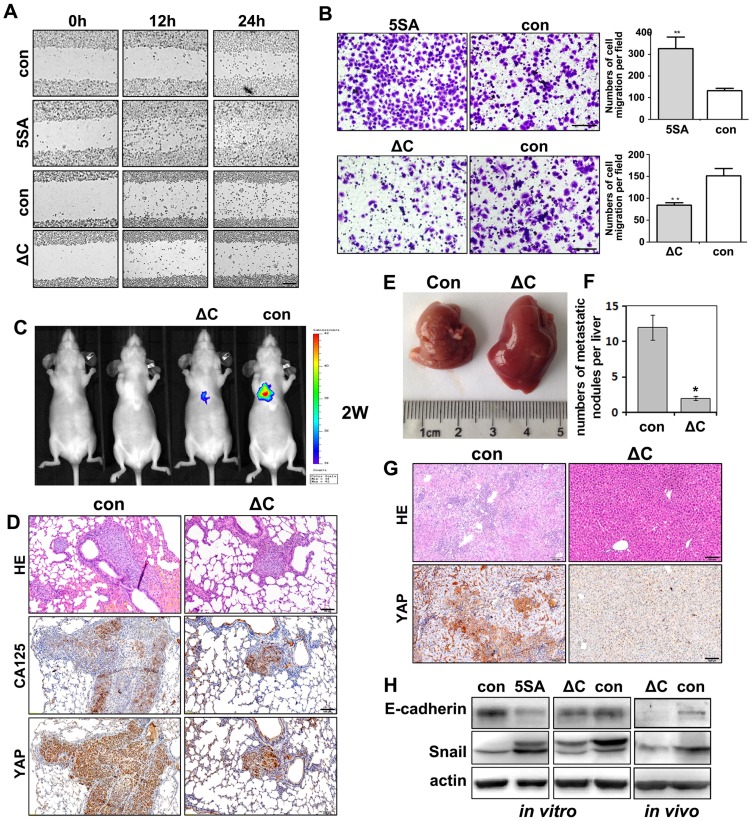
YAP promotes ovarian cancer cells migration and distant metastasis *in vitro* and *in vivo*. **A**. Wound-healing assay for the migration of YAP-5SA and YAP-5SA-ΔC expressing cells after culture for 12 and 24 hours. Scale bar  = 100 μM for all panels. **B**. Transwell assays for the migration of YAP-5SA and YAP-5SA-ΔC expressing cells. **, P<0.01. **C**. Bioluminescence imaging assays showing lung metastasis of control and YAP-5SA-ΔC expressing HO8910PM cells after they were injected into the caudal veins of nude mice. **D**. HE and IHC staining of lung metastasis sites shown in C. **E-F**: Images and statistical analysis results of liver metastasis by caudal vein injected HO8910 PM cells. **G**: HE and YAP IHC results of liver metastasis sites shown in E. **H**. Western blot results for EMT marker expressions in cultured YAP-5SA and YAP-5SA-ΔC expressing cells and *in vivo* xenografts derived from these cells.

We next investigated the effects of YAP activity on ovarian cancer cell migration. In both scratch assays (Figure 4A) and transwell assays (Figure 4B), YAP-5SA expressing cells migrated faster, whereas YAP-5SA-ΔC expressing cells migrated slower than the control cells.

In addition, we did *in vivo* experiments to test if YAP activity was required for ovarian cancer metastasis. We established YAP-5SA-ΔC- and luciferase-expressing stable cell lines using HO8910-PM cells, a human ovarian cancer cell line with a high metastatic capacity, and injected these cells into nude mice through their caudal veins (5×10^6^ cells/mouse). At two weeks after injection, mice were imaged for metastasis using an animal imaging system. As shown in Figure 4C–D, those xenografts derived from YAP-5SA-ΔC-expressing cells were smaller than those derived from control cells. IHC results showed that the xenografts were positive for CA125 and YAP, which confirmed their origins from the injected ovarian cancer cells. Significantly, post-inoculation metastases were detected in the livers of all 10 mice that were injected with control HO8910 cells, but only in 2 of 10 mice injected with YAP-5SA-ΔC-expressing cells (Figure 4E). In addition, YAP-5SA-ΔC-expressing cells formed far fewer metastatic nodules (YAP-positive) than control cells in the livers of injected mice (Figure 4F–G). These results suggested that decreased YAP activity retarded ovarian cancer cells metastasis *in vivo*.

An epithelial-to-mesenchymal transition (EMT) is thought to be an important process during the acquisition of those properties required for tumor metastasis. In both cultured ovarian cancer cells and in those isolated from *in vivo*, YAP-5SA promoted the expression of the EMT marker Snail and inhibited the expression of the epithelial marker E-cadherin. However, YAP-5SA-ΔC had the opposite effects (Figure 4H).

### High nuclear YAP and TAED4 levels are associated with a poor human ovarian cancer prognosis

**Figure 5:**
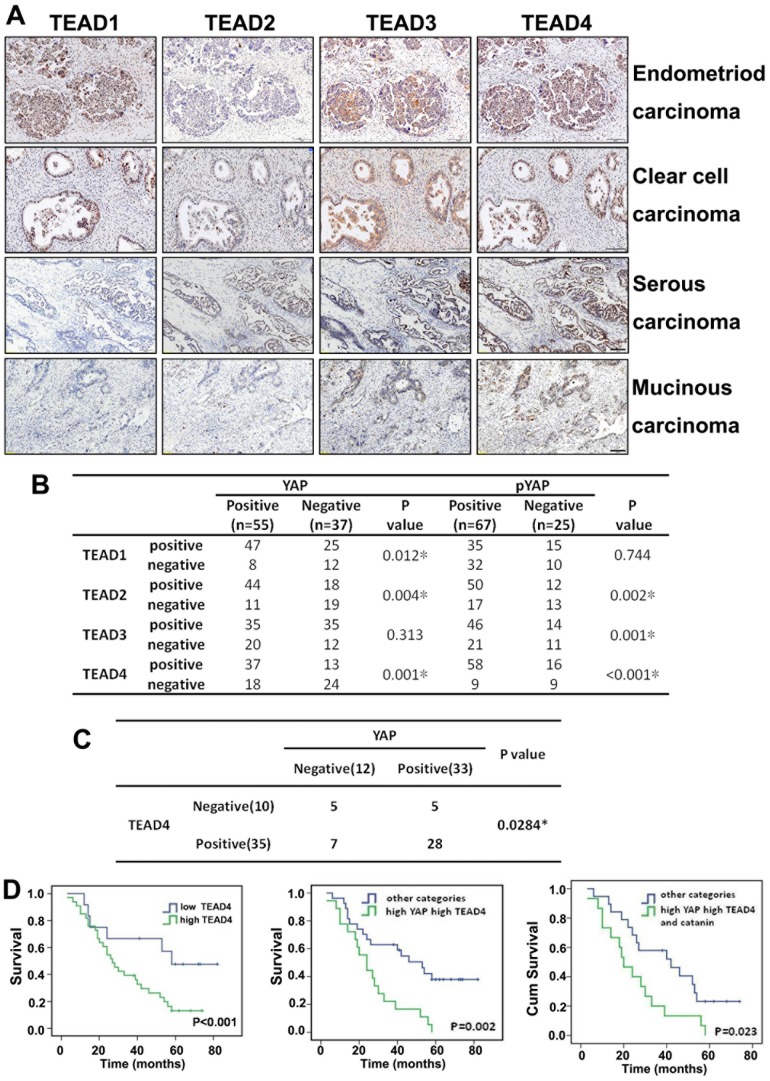
High YAP and TEAD4 co-expression levels are associated with a poor prognosis with primary human ovarian cancers. **A**. IHC results for TEADs expression in human ovarian cancer tissues. Scale bar  = 100 μM. **B**. Associations between YAP/pYAP expressions and TEAD expressions in an ovarian cancer tissue array. **C**. Associations between YAP and TEAD4 expressions in 45 ovarian cancer samples ranked by Kaplan-Meier survival estimates. **D**. YAP and TEAD4 co-expression is associated with a poor prognosis with human ovarian cancer. High TEAD expression versus low TEAD4 expression (left), high YAP expression combined with high TEAD4 expression versus other categories (middle), high YAP expression and high TEAD4 expression combined with high β-catanin versus other categories (right).

YAP does not have a DNA binding domain. It functions as a transcription co-activator by binding with TEAD family transcription factors (TEAD1-4). However, it has not been investigated if TEADs play any role in ovarian cancer prognosis. Thus, we investigated TEAD1-4 expression in human ovarian cancer samples. The expressions of the 4 subtypes of TEAD family proteins were investigated individually in an ovarian cancer tissue microarray by IHC (Figure 5A). Correlation analysis results showed that YAP and pYAP were positively correlated with TEAD1-4 expression, and were most closely correlated with TEAD4 expression (Figure 5B).An association analysis using 45 ovarian cancer patient samples was done in which we ranked Kaplan-Meier survival estimates. These results showed that YAP expression was positively correlated with TEAD4 expression (Figure 5C, P = 0.0284).

We further investigated if TEAD4 alone might be associated with ovarian cancer patient survival. Kaplan-Meier estimates and comparisons of disease-specific survival showed that high TEAD4 staining intensity was associated with poor patient survival (Figure 5D, left panel). The survival of patients with both high YAP and high TEAD4 expression was significantly worse than for the other categories (Figure 5D, middle panel; P = 0.002). In addition, the Kaplan-Meier estimate for combined high YAP/TEAD4 and the EMT marker β-catanin expression was also statistically significant (Figure 5D, right panel). Taken together, these results indicated that YAP and TEAD4 were two significant independent markers for poor patient survival and their combined evaluation might be a strong independent predictor of disease-specific survival for ovarian cancer patients.

## Discussion

YAP has been implicated as a possible oncogene with different subcellular localizations regulated by its upstream genes in the Hippo pathway. Increased YAP expression and a nuclear localization have been observed in multiple human cancers, including liver, colon, ovarian, and lung cancers [23]–[30]. In the present study, we showed that YAP protein levels were high in multiple ovarian cancer cell lines. More importantly, YAP was highly expressed in ovarian cancer samples but not in normal ovarian tissue, which suggested that YAP expression was directly related to ovarian cancer development or progression.

Previous studies also suggested that YAP might function as an oncogene in ovarian cancer [31]. However, none of these studies investigated possible associations between YAP/TEAD expression levels and ovarian cancer patient survival. In this study, we showed that high YAP expression in ovarian tumors was associated with shorter disease-specific survival for ovarian cancer patients, and that low YAP expression in ovarian tumors was associated with a longer disease-specific survival for these patients. Further, we found for the first time that TEAD4, a direct binding partner of YAP, was also closely associated with patient survival. The co-expression of YAP and TEAD4 in ovarian cancer tissues was even more dramatically associated with poor patient survival. Thus, high YAP/TEAD4 levels could be a predictor for determining ovarian cancer malignancy levels and for estimating the prognosis of these patients.

Primary epithelial ovarian cancers are usually treated with platinum-based drugs, such as cisplatin coupled with taxol. These drugs are administered either intravenously or intraperitoneally. Apoptotic and anti-apoptotic signals are the two aspects of cell cross-talk between cell survival and cell death pathways that determine the fates of cells in their reactions to exogenous circumstances. For tumors, drug-induced apoptosis is governed not only by the upregulation of apoptotic factors or tumor suppressors, but also by the modulation of cell survival factors. In the present study, we showed that up- and down-regulation of YAP activity could modulate the proliferation, colony formation and migration capabilities, and the resistance to chemotherapeutic agents by ovarian cancer cell lines. A constitutively active YAP mutant increased the resistance to drug-induced apoptosis by ovarian cancer cells, whereas dominant negative YAP restored drug sensitivity in Cisplatin-resistant ovarian cancer cells.

The precise mechanisms by which YAP enhances drug resistance remain unclear. Several studies focused on the PI3K/Akt pathway for chemo-resistance in ovarian cancers and showed that Cisplatin could upregulate p53 and induce apoptosis in these cancer cells after expression of dominant negative AKT, which suggested that cisplatin-mediated p53 upregulation was opposed by AKT. The PI3K/AKT pathway might also participate in YAP-mediated drug resistance, as it has been clearly shown that YAP was involved in cross-talk with PI3K pathways and facilitated AKT activation in other cell types [32], [33]. Previous studies also showed that hyperactivation of the PI3K pathway initiated ovarian cancer development in mouse models. The interaction of these two pathways needs to be further investigated in ovarian cancer cells.

It has been hypothesized that cancer cells lose their epithelial characteristics and acquire certain mesenchymal properties that promote extracellular environment invasion and distant metastasis in an EMT-like process. Epithelial markers that are down-regulated during this transition include E-cadherin, cytokeratins, ZO-1 and others. Hippo signaling pathway components are required for E-cadherin-dependent contact inhibition of proliferation. Knockdown of Hippo signaling components or YAP overexpression inhibits the decreased cell proliferation caused by E-cadherin homophilic binding at the cell surface [34].

In our study, we found that YAP could enhance the migration of ovarian cancer cell lines. We also found that the expression of EMT markers was modulated by YAP activity in tumor tissues of nude mice and confirmed that YAP enhanced ovarian cancer cell metastasis *in vivo*. Although YAP expression combined with EMT marker expressions was associated with patient prognosis, E-cadherin and other EMT genes alone were not associated with patient survival (data not shown). Thus, E-cadherin and other EMT genes were not independent predictors for ovarian cancer risk prediction. If YAP is taken into account, then EMT markers might be a predictor of ovarian cancer risk.

Previous studies suggested that YAP was an independent prognostic marker for poor survival with ovarian cancer and other carcinomas [9], [30]. Our study also confirmed this using survival analysis. In our data set, YAP was positively associated with patient prognosis, whereas its cytoplasmic distribution and pYAP were not indicators of survival. Patients with high nuclear YAP (nYAP) expression and low cytoplasmic YAP (cYAP) expression had a nearly eight times greater risk of death from this disease than other patients. The nYAP/cYAP pattern might reflect YAP activity in a tumor: YAP has transcription-promoting activity only when it is localized in the nucleus and is presumably transcriptionally inactive when it is retained in the cytoplasm and is phosphorylated at S127 by LATS1/2.

Several studies found that increased YAP activity could promote TEAD-dependent transcription in a manner that depended on the TEAD-interaction domain of YAP [14], [35]–[38]. Thus, we investigated whether increased YAP activity was associated with increased TEAD expression in ovarian cancer and its relationship with patient survival. Our immunostaining results showed that YAP was positively correlated with TEAD family proteins' expression, except for TEAD3. The correlation with TEAD4 was the highest among the four TEAD family genes.

We categorized TEAD4-expressing ovarian cancer samples into those with weak and strong expressions and compared these with patient prognosis. These results showed that strong YAP and TEAD4 expression was a predictor of reduced ovarian cancer survival. Thus, YAP and TEAD4 might biochemically function together in ovarian cancer progression, and YAP-TEAD4 co-expression may be important for assessing ovarian cancer patient outcomes.
