# Correction: YAP Promotes Ovarian Cancer Cell Tumorigenesis and Is Indicative of a Poor Prognosis for Ovarian Cancer Patients

**DOI:** 10.1371/journal.pone.0152712

**Published:** 2016-03-25

**Authors:** Yan Xia, Ting Chang, Yingmei Wang, Yixiong Liu, Wenhui Li, Ming Li, Heng-Yu Fan

In Panel A of Fig 2, the fourth lane is incorrectly labelled “C12.” This lane should be labelled “C13”. Please see the corrected Fig 2 here.

**Fig 2:**
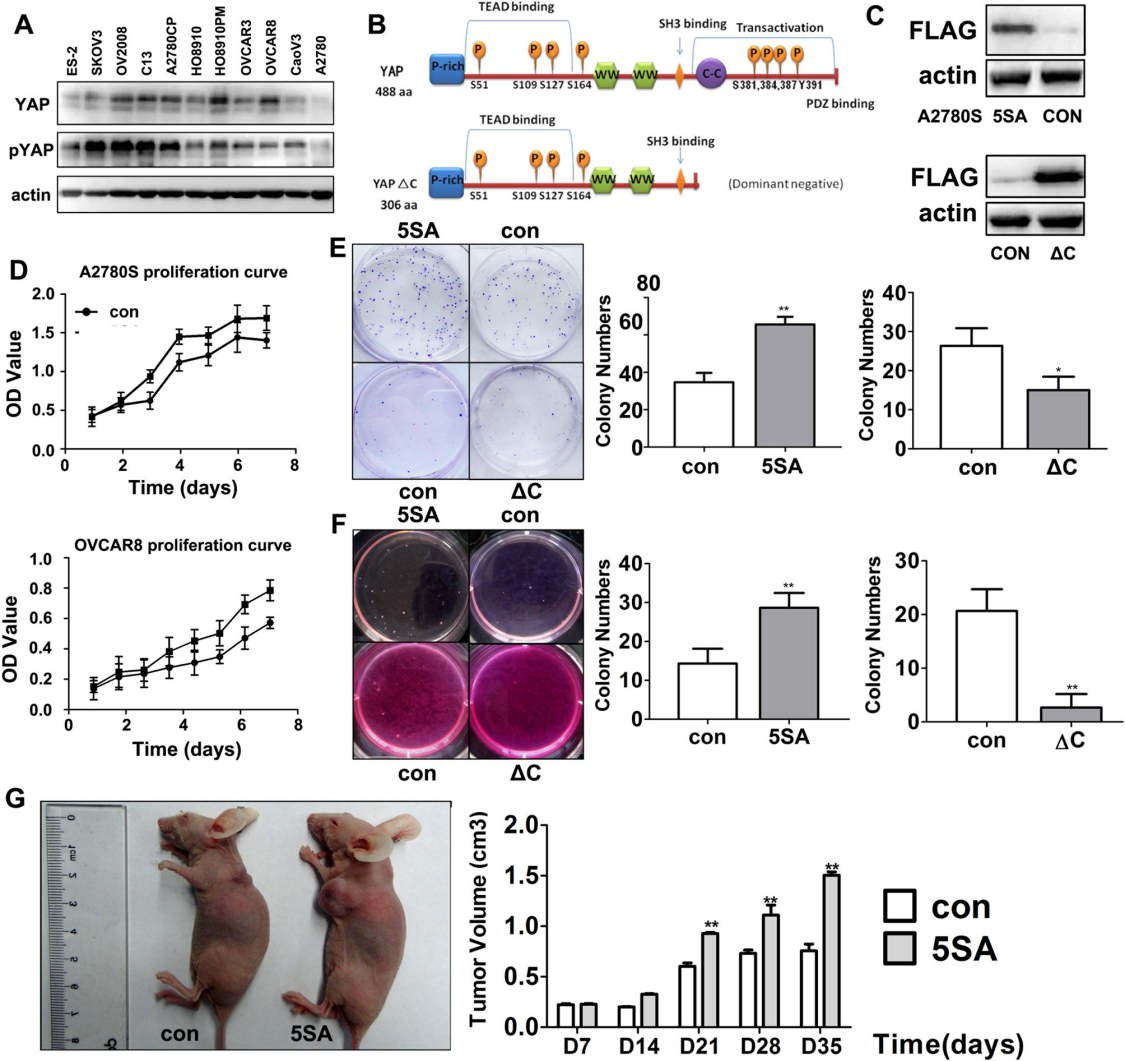
YAP promotes human ovarian cancer cell proliferation and tumorigenesis. A: Western blotting results for endogenous YAP expression in 11 ovarian cancer cell lines. B: Diagram of the main functional structural domains and phosphorylation sites in human YAP (full length and C-terminal deleted, YAP-ΔC, forms). C: Western blotting results for the expression of FLAG-tagged YAP-5SA and YAP-5SA-ΔC in established stable cell lines. D: Growth curves for cells that stably expressed YAP-5SA (upper panel) and YAP-5SA-ΔC (lower panel) and their control cells during a 7-day culture period. E: Images and quantitative results for flat plate colony formation assays. Cells that expressed YAP-5SA or YAP-5SA-ΔC and their control cells were cultured in 6-well plates for 2 weeks. F: Images and quantitative results for colonies grown in soft agar. Cells that expressed YAP-5SA or YAP-5SA-ΔC and their control cells were seeded in soft agar for 3 weeks. G: Images and quantitative results for xenografts grown in nude mice. Mice were subcutaneously injected with YAP-5SA expressing and control cells. Each point is the mean of 3 experiments. Error bars represent s.d. 's; n = 5. Statistically significant differences as compared with a control as determined by Student's t-test are denoted by *(P<0.05) or **(P<0.01).

There is an error in the caption for Fig 3, “YAP enhances chemotherapeutic drug resistance by ovarian cancer cells,” panels C-E. Please see the complete, correct Fig 3 caption here.

**Fig 3:**
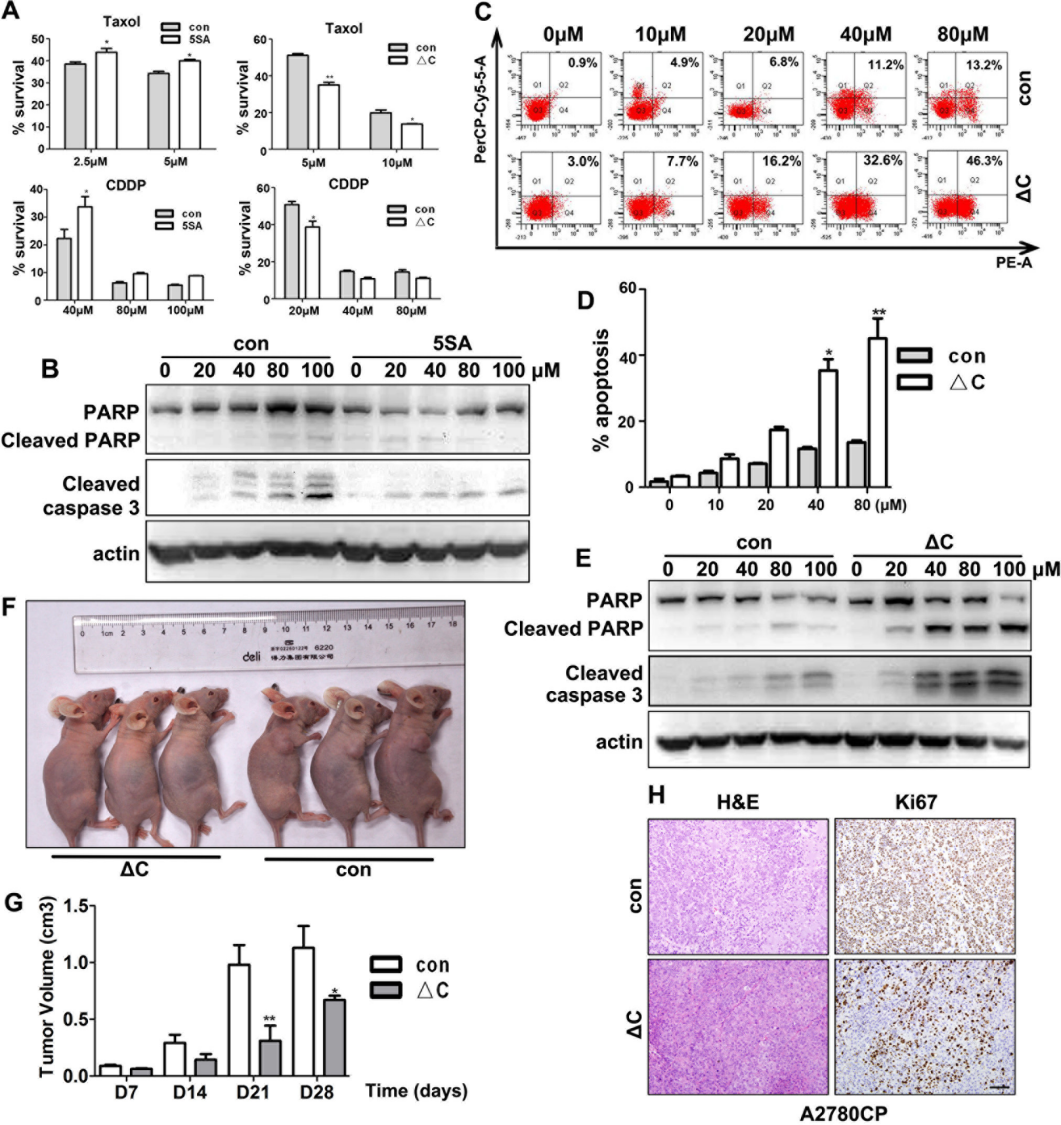
YAP enhances chemotherapeutic drug resistance by ovarian cancer cells. A: Viability of YAP-5SA and YAP-5SA-ΔC expressing cells after treatment with Taxol or CDDP, as assessed by MTT assay. B: Western blotting results for the apoptosis markers cleaved caspase 3 and PARP in YAP-5SA expressing and control cells after treatment with the indicated doses of CDDP for 48 h. C-D: Flow cytometry results for apoptosis of A2780CP cells with or without YAP-5SA-DC transfection and after treatment with different doses of CDDP for 48 h. E: Western blotting results for cleaved caspase 3 and PARP in YAP-5SA-DC expressing and control cells after treatment with the indicated doses of CDDP for 48 h. F-G: Images and quantitative results for *in vivo* tumorigenic capacity of A2780CP cells with or without YAP-5SA-ΔC expression. Nude mice were injected with CDDP through a caudal vein once each week for four weeks after tumor xenografts reached 5 mm in diameter. H: IHC results for the proliferation marker Ki67 on the indicated tumor tissue sections. Scale bar = 100 μM.
